# Attenuation of highly pathogenic avian influenza A(H5N1) viruses in Indonesia following the reassortment and acquisition of genes from low pathogenicity avian influenza A virus progenitors

**DOI:** 10.1038/s41426-018-0147-5

**Published:** 2018-08-22

**Authors:** Ni Luh Putu Indi Dharmayanti, Sharmi W. Thor, Natosha Zanders, Risza Hartawan, Atik Ratnawati, Yunho Jang, Marisela Rodriguez, David L. Suarez, Gina Samaan, Pudjiatmoko, C. Todd Davis

**Affiliations:** 1 Indonesian Research Center for Veterinary Science, Bogor, Indonesia; 2 Influenza Division, Centers for Disease Control and Prevention, Atlanta, GA USA; 3 Southeast Poultry Research Laboratory, USDA, Athens, GA USA; 4 Australian National University, Canberra, Australia; 5 Ministry of Agriculture, Jakarta, Indonesia

## Abstract

The highly pathogenic avian influenza (HPAI) A(H5N1) virus is endemic in Indonesian poultry and has caused sporadic human infection in Indonesia since 2005. Surveillance of H5N1 viruses in live bird markets (LBMs) during 2012 and 2013 was carried out to provide epidemiologic and virologic information regarding viral circulation and the risk of human exposure. Real-time RT-PCR of avian cloacal swabs and environmental samples revealed influenza A-positive specimens, which were then subjected to virus isolation and genomic sequencing. Genetic analysis of specimens collected at multiple LBMs in Indonesia identified both low pathogenicity avian influenza (LPAI) A(H3N8) and HPAI A(H5N1) viruses belonging to clade 2.1.3.2a. Comparison of internal gene segments among the LPAI and HPAI viruses revealed that the latter had acquired the PB2, PB1, and NS genes from LPAI progenitors and other viruses containing a wild type (wt) genomic constellation. Comparison of murine infectivity of the LPAI A(H3N8), wt HPAI A(H5N1) and reassortant HPAI A(H5N1) viruses showed that the acquisition of LPAI internal genes attenuated the reassortant HPAI virus, producing a mouse infectivity/virulence phenotype comparable to that of the LPAI virus. Comparison of molecular markers in each viral gene segment suggested that mutations in PB2 and NS1 may facilitate attenuation. The discovery of an attenuated HPAI A(H5N1) virus in mice that resulted from reassortment may have implications for the capability of these viruses to transmit and cause disease. In addition, surveillance suggests that LBMs in Indonesia may play a role in the generation of reassortant A(H5) viruses and should be monitored.

## Introduction

The highly pathogenic avian influenza (HPAI) A(H5N1) virus, which was first introduced into Indonesia in 2003, is a cause of economically devastating disease in poultry. Since being introduced, the virus has become endemic in the country, causing recurring outbreaks that have resulted in the death of millions of birds due to either infection or the culling of animals. Despite the implementation of vaccination programs in many regions, the emergence of antigenic drift variants and the high economic costs associated with vaccination both present constant challenges for the control of the spread of influenza^1,2^. Human infection with HPAI A(H5N1) virus primarily occurs via poultry-to-human transmission, with very few reports of human-to-human transmission^3^. The risk factors for human infection include exposure to infected poultry^4^ and, as illustrated by recent cases of influenza in humans in Indonesia and many other countries, exposure to live bird markets (LBMs), which still play an important role in the marketing of poultry to consumers in Indonesia^5^. As of July 2018, Indonesia had the second highest number of influenza cases that were reported to the World Health Organization (WHO), with a case fatality rate of 84% (200 confirmed cases and 168 deaths)^6^. A(H5N1) was first identified in Indonesian poultry on the island of Java in December 2003^7^. The virus quickly spread and, by December 2007, outbreaks in poultry had been confirmed in the majority of Indonesia’s provinces^8^. Viral evolution has resulted in the designation of genetically and antigenically distinct H5 phylogenetic clusters, termed clades, which are numbered 0–9. The majority of A(H5N1) viruses detected in Indonesia most likely descended from a single ancestor derived from clade 2.1 HA^9^, but as the restrictions on the movement of poultry increased, the introduced viruses evolved independently into those making up the second, third, and fourth order clades (2.1.1, 2.1.2, 2.1.3, 2.1.3.1, 2.1.3.2, 2.1.3.2a, and 2.1.3.2b) on the thousands of islands that comprise the country of Indonesia^10,11^.

Indonesia has a multifaceted surveillance network in place that links veterinary and human health organizations with epidemiological and laboratory units^5^. This network is charged with monitoring the areas where human infection with A(H5N1) is likely, including live bird markets. In this study, we report on the viruses that were detected during outbreak investigations associated with human influenza A(H5N1) virus cases as well as during routine influenza virus surveillance in birds. Surveillance of A(H5N1) viruses in live bird markets was conducted throughout Indonesia during 2012 and 2013. The viruses that were characterized were detected in three provinces in Indonesia. Two specimens were obtained during the investigations of confirmed H5N1-human cases (in Bekasi and Karawang in the West Java province), one specimen was obtained during an investigation of a suspected human case in Bengkulu, and two other specimens were from districts in East Java, Gresik and Surabaya that had not been visited for surveillance purposes for at least two years. Avian and environmental samples were analyzed for the presence of influenza A virus. Analysis of the HA gene revealed five viruses were HPAI A(H5N1), which were phylogenetically related to other clade 2.1.3.2a viruses, and one virus was LPAI A(H3N8). Examination of the NA and internal gene segments among the isolated LPAI and HPAI viruses revealed that several of the HPAI A(H5N1) clade 2.1.3.2a viruses contained gene segments derived from an LPAI virus, indicating recent reassortment. The reassortant viruses possessed PB2, PB1, and NS genes obtained from an LPAI virus, while the surface glycoproteins (HA and NA) and other internal genes (PA, NP, and M) came from a virus of HPAI A(H5N1) lineage. The close genetic relationship of the LPAI genes detected in the reassortant A(H5) virus to that of the LPAI A(H3N8) virus isolated during this study suggests the possible origin of the reassortant genes. The infectivity and virulence in mice, as well as the replication kinetics in chicken eggs and cells, were compared among the wt A(H5N1), reassortant A(H5N1) and LPAI A(H3N8) viruses.

## Materials and methods

### Virus collection, isolation, and sequencing

The Indonesian Research Center for Veterinary Science (IRCVS) in Bogor, Indonesia, collected and analyzed samples obtained during investigations associated with one suspected and two confirmed human influenza A(H5N1) virus infection reports, as well as from the surveillance of active influenza viruses circulating in live bird markets. The investigations of the human cases included the collection of poultry cloacal swabs and environmental samples from backyard flocks and LBMs visited by the patient or in the vicinity of the home of the patient. Sick birds were prioritized for sampling. Environmental surface swabs from chopping boards, knives, meat display tables and feces from cages were also collected. In addition, active surveillance of the A(H5N1) virus was conducted in multiple LBMs by IRCVS. Environmental swabs from markets were collected and tested individually, while the bird cloacal swabs were pooled in groups of five, as shown in Table S1. Real-time RT-PCR, targeting the influenza A virus matrix (M) gene, was conducted to identify Influenza A. The positive specimens were inoculated into 10-11 day old embryonated chicken eggs. After incubation, the allantoic fluid was harvested and the viral RNA was isolated using the Qiagen RNeasy extraction kit (Qiagen). The approval of the CDC Institutional Animal Care and Use Committee (IACUC) was not required in order to conduct virus propagation in embryonated chicken eggs, as all of the eggs were destroyed prior to hatching. All work was carried out according to guidance from the Office of Laboratory Animal Welfare at the National Institutes of Health, which is responsible for the implementation of the PHS Policy Animal Welfare Act (7 U.S.C. Sections 2131-2159) and the Public Health Service Policy on the Humane Care and Use of Laboratory Animals (http://grants.nih.gov/grants/olaw/faqs.htm#App_4).

The viral RNA that was purified from each isolate was tested using the CDC Influenza Virus Real-time RT-PCR Influenza A(H5N1) (Asian Lineage) Subtyping Panel kit (http://www.cdc.gov/flu/clsis). Additional primers and probes (available on request) were used to detect the LPAI subtypes and to screen the H5-positive samples for the presence of mixed infections. The samples were also tested for the Eurasian-lineage A(H7), A(H9), and Newcastle disease (NDV) viruses. All rRT-PCRs were performed using the Invitrogen Superscript III Platinum One-Step qRT-PCR kit (Invitrogen; Carlsbad, California). The gene expression and comparative threshold cycle values were analyzed using the Stratagene Mx3005 detection system (data not shown). The purification of four virus samples via egg limiting dilution in the presence of NDV-neutralizing antibodies was necessary to eliminate NDV from the samples. Following purification, all of the isolates were inoculated into 10–11 day old embryonated chicken eggs, and the allantoic fluid was harvested for use in the preparation of viral stocks. The allantoic fluid samples were clarified by centrifugation, and the aliquots were stored at −70 °C until further use. The stocks were titrated in MDCK cells using standard plaque assay methods for the determination of the PFU titer and/or for the calculation of the 50% egg infectious dose (EID50/mL), both using standard methods^12^. All of the experiments were performed in biosafety level 3 containment laboratories with enhancements as required by the Animal and Plant Health Inspection Service (US Department of Agriculture) and the National Select Agent Program (Department of Health and Human Services). Influenza viral RNA was amplified using the Access Quick One-Step RT-PCR kit (Promega) using H5N1 and H3N8 HA- and NA-specific primers (primer sequences are available upon request). The internal genes were amplified using universal influenza A-specific primers designed to amplify internal genes (primer sequences are available upon request). The sequences of the PCR amplicons were determined via Sanger sequencing using an Applied Biosystems 3730xl system with v3.0 cycle sequencing dye terminator chemistry (Applied Biosystems, Foster City, California). The full-length open reading frames (ORFs) for each gene segment obtained from the virus stocks were generated using Sequencher 5.0 (Gene Codes; Ann Arbor, Michigan). The exclusion of specimens containing other possible subtypes was performed by rRT-PCR, as described above, using subtype-specific assays to rule out the presence of mixed infections. The gene sequences that were obtained were submitted to the GISAID database with accession numbers as follows:

(A/Muscovy duck/EastJava/SB29/2012) EPI_ISL_284315: (PB2) EPI109573, (PB1) EPI1095733, (PA) EPI1095731, (HA) EPI1095735, (NP) EPI1095728, (NA) EPI1095734, (M) EPI1095730, (NS) EPI1095729;

(A/Muscovy duck/EastJava/LM47/2012) EPI_ISL_284316: (PB2) EPI1095740, (PB1) EPI1095741, (PA) EPI1095739, (HA) EPI1095743, (NP) EPI1095736, (NA) EPI1095742, (M) EPI1095738, (NS) EPI1095737;

(A/chicken/WestJava/KRW54/2012) EPI_ISL_284318: (PB2) EPI1095756, (PB1) 2014700454, (PA) EPI1095755, (HA) EPI1095759, (NP) EPI1095752, (NA) EPI1095758, (M) EPI1095754, (NS) EPI1095753;

(A/chicken/EastJava/BP21/2012) EPI_ISL_284317: (PB2) EPI1095748, (PB1) EPI1095749, (PA) EPI1095747, (HA) EPI1095751, (NP) EPI1095744, (NA) EPI1095750, (M) EPI1095746, (NS) EPI1095745;

(A/environment/WestJava/BKSI37/2013) EPI_ISL_284319: (PB2) EPI1095764, (PB1) EPI1095765, (PA) EPI1095763, (HA) EPI1095767, (NP) EPI1095760, (NA) EPI1095766, (M) EPI1095762, (NS) EPI1095761;

(A/chicken/Bengulu/RJL24/2013) EPI_ISL_284320: (PB2) EPI1095772, (PB1) EPI1095773, (PA) EPI1095771, (HA) EPI1095775, (NP) EPI1095768, (NA) EPI1095774, (M) EPI1095770, (NS) EPI1095769.

### Phylogenetic analysis

The eight viral genes from each isolate were phylogenetically analyzed based on datasets of relevant viral sequences from Indonesia and other Asian or European countries that were identified by BLAST analysis of the avian and environmental samples using both the GISAID (http://platform.gisaid.org) and the NCBI databases (http://www.ncbi.nlm.nih.gov). Over 90 sequences per gene segment were aligned using the Muscle algorithm (http://www.drive5.com/muscle/). The evolutionary histories of the isolated viruses were inferred using a maximum likelihood calculation based on the Tamura-Nei model using the MEGA7 software package (www.megasoftware.net). The reliability of each phylogenetic analysis was tested using a bootstrap analysis with 1000 bootstrap replicates. The phylogenetic trees were either rooted to A/goose/Guangdong/1/1996 or were midpoint rooted. The gene lineages for HA were defined using the criteria described by the WHO/OIE/FAO H5N1 Evolution Working Group^11,13,14^. For molecular analysis of the viral proteins, the full-length ORF sequences encoding the mature HA, NA and internal gene-derived proteins were used. The amino acid sequences corresponding to each viral protein were analyzed for mutations that could putatively confer the observed viral phenotypes by comparing them to sequences in the CDC H5N1 Genetic Changes Inventory (https://www.cdc.gov/flu/avianflu/h5n1/inventory.htm) as well as similar or identical sequences containing mutations that were previously reported.

### Determination of mouse infectivity and virulence

All mouse procedures were approved by the IACUC of the Centers for Disease Control and Prevention and performed in a BSL-3 facility with enhancements. Female BALB/c mice, 6–8 weeks old (James River), were lightly anesthetized using isoflurane bioinhalation prior to intranasal (i.n.) infection with fifty microliters of infectious virus per nare. Studies were performed with one representative wt HPAI A(H5N1) virus (A/environment/West Java/BKSI37/2013), one HPAI reassortant A(H5N1) virus (A/chicken/East Java/BP21/2012), and one LPAI A(H3N8) virus (A/environment/West Java/KRW54/2012). Each virus was diluted in PBS to provide a dose between 10^1^ EID_50_ and 10^4^ EID_50_, with the exception of the LPAI A(H3N8) virus, which was administered with a dose between 10^1^ EID_50_ and 10^5^ EID_50_. The 50% mouse infectious dose (MID_50_) and 50% lethal dose (LD_50_) were determined based on the inoculation of 8 mice per dosage group. Four days post-infection, tissues (nose, lung, spleen, and brain) were collected from 3 mice selected at random from each dosage group. The tissues were immediately frozen and stored at −70 °C until they were processed. The frozen tissues were later thawed, homogenized in 1 ml of cold PBS supplemented with 10% penicillin-streptomycin and 10% gentamicin, and clarified by centrifugation at 3000x*g* for 1 min at 4 °C. The viral infectivity of the tissue homogenate was determined using a plaque assay in MDCK cells in order to determine the positive versus the negative tissues for the purpose of the MID_50_ calculations. The specific viral infectivity titers in the nose, lung, spleen and brain at the 10^3^ dosage were determined using a plaque assay in MDCK cells in order to compare the viral titers in tissues at the median dose. The remaining mice in each group were monitored daily for clinical signs of infection and changes in body weight. Any mice exhibiting a clinical score of 4 (with ruffled fur, a hunched posture, reduced locomotion, or a lack of response) or having lost more than 25% of the pre-inoculation body weight were humanely euthanized. All of the surviving mice were humanely euthanized at 14 days post-infection. The MID_50_ and LD_50_ values were calculated using the method of Reed and Muench^12^.

### Replication kinetics in embryonated chicken eggs and chicken embryo fibroblast cells

The replication capacity of the viruses tested during the mouse studies were assessed in avian substrates in vitro by measuring post-infection titers in both embryonated chicken eggs (ECEs) and chicken embryo fibroblast (CEF) cells. The allantoic fluid from inoculated ECEs and supernatants from inoculated CEF cells were collected at 8, 12, 24, 48, and 72 h post-inoculation. Briefly, three ECEs and three wells containing CEF cells were inoculated at an MOI of 0.01 for the CEFs, with a comparable dilution inoculated into the ECEs. The infectivity titers (calculated as EID_50_/ml) were determined using the method of Reed and Meunch^12^. The statistical analysis and the determination of significant differences between time points were carried out using the GraphPad Prism software package (https://www.graphpad.com).

## Results

### Epidemiology of human cases and samples collected

During this study, 3 viruses were identified from human case outbreak investigations, as shown in Table S1. The first, a low pathogenicity A(H3N8) virus (A/environment/West Java/KRW54/2012), was collected from an LBM during an investigation in July 2012 in West Java Province. An 8-year-old female visited a live bird market 6 days prior to the onset of illness, on 18 June 2012. She was confirmed to be infected with the A(H5N1) virus on 29 June and died on 4 July. The second virus, A/chicken/Bengkulu/RJL24/2013, was associated with a suspected human A(H5N1) infection in Bengkulu Province. A 39-year-old male bought chickens from a live bird market, which subsequently died at the patient’s home on 28 February 2013. He developed influenza-like illness 4 days later. The specimens collected from the patient’s home on 6 March were negative for influenza virus. However, one pool of samples collected from spent hens at an LBM in the patient’s neighborhood tested positive for A(H5N1). The third virus, isolated from an A(H5N1)-positive environmental sample (A/environment/West Java/BKSI37/2013), was obtained during an investigation in West Java Province. A 2.5-year-old male visited a live bird market two days before the onset of illness on 10 June 2013. He was confirmed to be infected with the A(H5N1) virus on 19 June and died the same day. The pooled environmental specimens were composed of chopping board swabs obtained from the LBM that was visited by the patient who later tested positive for the A(H5N1) virus, suggesting the potential source of the infection.

An additional three viruses, as shown in Table S1, were identified during routine avian influenza virus surveillance conducted at live bird markets and poultry collection yards in three districts, Gresik, Surabaya and Lamongan, in East Java Province in November 2012. In Gresik, three environmental swabs and one pool of three bird samples from a single live bird market were positive for the A(H5N1) virus. The isolate from Gresik was obtained from a bird (A/chicken/East Java/BP21/2012). In Surabaya, one pool of cloacal swabs collected from geese tested positive for the A(H5N1) virus (A/muscovy duck/East Java/SB29/2012). In Lamongan, one environmental swab from a live bird market was A(H5N1) positive, and one pool of Muscovy duck cloacal swabs collected from a bird collection yard tested positive (A/muscovy duck/East Java/LM47/2012).

### Phylogeny of Indonesian viruses

**Fig. 1:**
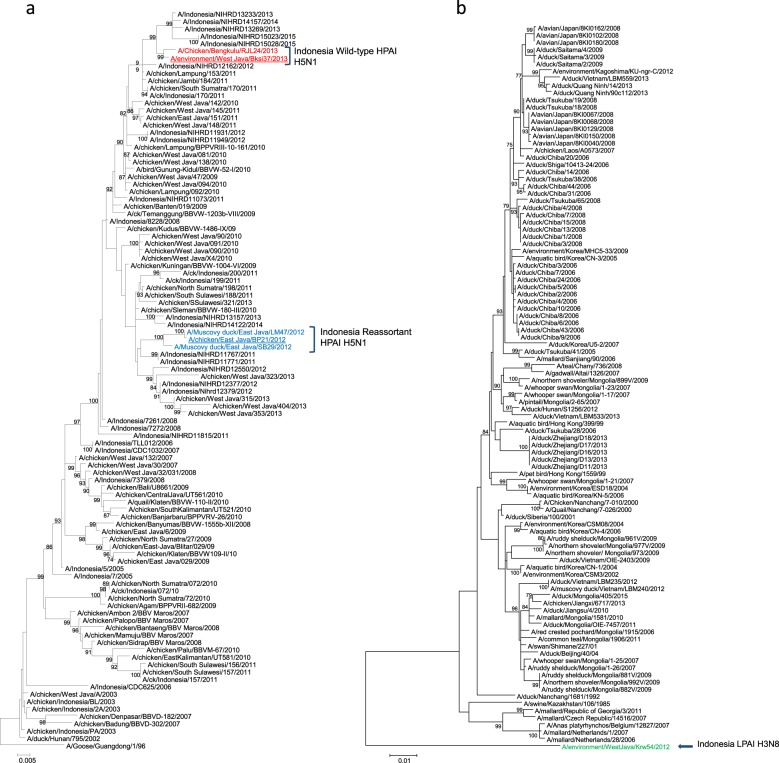
Phylogenetic tree of (**a**) HPAI A(H5N1) clade 2.1.3.2a HA genes and **b** LPAI A(H3) HA genes. Viruses characterized herein are identified as follows: Indonesia H5N1 strain names are colored red, Indonesia reassortant H5N1 strain names are colored blue, Indonesia LPAI H3N8 strain name is colored green, and all viruses further utilized for in vivo studies are underlined. Bootstrap values calculated following 1000 replicates are shown above each branch

The HA clade of each of the Indonesian viruses was determined using phylogenetic analysis. Five of the six viruses analyzed were found to share a common ancestor, A/goose/Guangdong/1996, and belonged to clade 2.1.3.2a (which includes A/muscovy duck/East Java/SB29/2012, A/muscovy duck/East Java/LM47/2012, A/chicken/East Java/BP21/2012, A/environment/West Java/BKSI37/2013, and A/Chicken/Bengkulu/RJL24/2013). The HA gene from each of these viruses was grouped spatially and temporally with that from related HPAI A(H5N1) Indonesian viruses that had bootstrap values greater than 99%, as shown in Fig. 1a. The H3 HA gene from A/environment/West Java/KRW54/2012 could not be grouped with that from the other Indonesian viruses that were included in the analysis; therefore, this virus was classified as part of another phylogenetic tree containing the other LPAI H3 viruses within the Eurasian lineage (Fig. 1b). The BLAST analysis based on the GISAID database demonstrated that this virus was most closely related to an LPAI A(H3N8) virus, A/duck/Siberia/100/2001, sharing only a 90% nucleotide identity. A/environment/West Java/KRW54/2012 thus appears to be an outlier within the HA phylogeny due to the lack of related viruses that can be found in public databases. The NA gene from each of the HPAI A(H5N1) viruses exhibited a similar phylogeny to that of the HA gene, clustering with other A(H5N1) viruses from Indonesia obtained from similar geographic locations and on similar collection dates (Figure S1 (a)). The NA from A/environment/West Java/KRW54/2012 was found to be most closely related to that from an LPAI H6N8 A/pintail duck/Alberta/628/1979 virus, sharing a 93% nucleotide identity according to a BLAST analysis based on the GISAID database (Figure S1(b)). The analysis of the internal gene sequences revealed evidence of reassortment (Figure S2(a–f)). The phylogenies of the HPAI A(H5N1) virus PA, NP, and M gene sequences were similar to those of the HPAI A(H5N1) HA and NA genes; the viruses were clustered together spatially and temporally, with no evidence of reassortment (Figure S2). However, the phylogenetic analysis of the PB2, PB1, and NS gene sequences from the East Java viruses (A/muscovy duck/East Java/SB29/2012, A/muscovy duck/East Java/LM47/2012, and A/chicken/East Java/BP21/2012) revealed that these viruses were clustered together to form an outgroup in relation to the HPAI A(H5N1) viruses (Figure S2(a–d, f)). These three genes were clustered most closely with those from the LPAI A(H3N8) virus A/environment/West Java/KRW54/2012, providing evidence that reassortment between HPAI A(H5N1) clade 2.1.3.2a viruses and LPAI A viral progenitors had occurred. BLAST analysis based on the GISAID database determined that each of these genes was most closely related to those from other avian LPAI viruses (≥91%; Table 1).

**Table 1:** Genome constellations of avian influenza A viruses collected from live bird markets and human case investigations in Indonesia

Strain name	LBM district (province)	Genotype	HA	NA	PB2	PB1	PA	NP	M	NS
A/Environment/West Java/Bksi37/2013^a^	Bekasi (West Java)	HPAI A (H5N1) wt	A/chicken/Karimun/BPPVII 719-11/2010 (H5N1) 99.0%	A/Indonesia/CDC938/2006 (H5N1) 95.8%	A/chicken/Majalengka/08160070-001/2016 (H5N1) 97.7%	A/Indonesia/CDC1046T/2007 (H5N1) 96.7%	A/quail/Deliserdang/01160025/2016 (H5N1) 97.9	A/Indonesia/524 H/2006 (H5N1) 98.1%	A/eagle/Jakarta Timur/20616-III/2016 (H5N1) 98.2%	A/quail/Deliserdang/01160025/2016 (H5N1) 98.2%
A/chicken/Bengkulu/RJL24/2013^a^	Rejang Lebung (Bengkulu)	HPAI A (H5N1) wt	A/chicken/Karimun/BPPVII 719-11/2010 (H5N1) 98.8%	A/Indonesia/CDC1032/2007 (H5N1) 95.7%	A/chicken/Majalengka/08160070-001/2016 (H5N1) 97.8%	A/Indonesia/CDC1046T/2007 (H5N1) 96.9%	A/quail/Deliserdang/01160025/2016 (H5N1) 97.6%	A/quail/Deliserdang/01160025/2016 (H5N1) 98.2%	A/eagle/Jakarta Timur/20616-III/2016 (H5N1) 98.5%	A/quail/Deliserdang/01160025/2016 (H5N1) 98.5%
A/Muscovy duck/East Java/SB29/2012^b^	Surabaya (East Java)	HPAI A (H5N1) reassortant	A/chicken/Sukoharjo/BBVW-1148-07/2011 (H5N1) 99.1%	A/chicken/West Java Tangerang/PTB6/2008 H5N1 (99%)	A/duck/Mongolia/47/2001 (H7N1) 91.0%	A/goose/Eastern China/17/2010 (H6N6) 91.1%	A/Indonesia/CDC1031T/2007 (H5N1) 97.7%	A/Indonesia/524 H/2006 (H5N1) 98.1%	A/chicken/West Java/Smi-Acul/2008 (H5N197.7%	A/mallard duck/Netherlands/08/2000 (H3N8) 95.1%
A/Muscovy Duck/East Java/LM47/2012^b^	Lamongan (East Java)	HPAI A (H5N1) reassortant	A/chicken/Sukoharjo/BBVW-1148-07/2011 (H5N1) 98.5%	A/chicken/West Java Tangerang/PTB6/2008 H5N1 (99%)	A/duck/Mongolia/47/2001 (H7N1) 93.6%	A/goose/Eastern China/17/2010 (H6N6) (91.0%)	A/Indonesia/CDC1031T/2007 (H5N1) 97.8%	A/Indonesia/524 H/2006 (H5N1) 98.1%	A/chicken/West Java/Smi-Acul/2008 H5N1 (99%)	A/mallard duck/Netherlands/08/2000 (H3N2) 95.1%
A/Chicken/East Java/BP21/2012^b^	Gresik (East Java)	HPAI A (H5N1) reassortant	A/chicken/Sukoharjo/BBVW-1148-07/2011 (H5N1) 98.5%	A/chicken/West JavaTangerang/PTB6/2008 (H5N1) 97.2%	A/duck/Mongolia/47/2001 (H7N1) (92.8%)	A/goose/Eastern China/17/2010 (H6N6) 90.5	A/Indonesia/CDC1031T/2007 (H5N1) 97.2%	A/Indonesia/524 H/2006 (H5N1) 97.8%	A/chicken/West Java/Smi-Acul/2008 (H5N1) 97.6%	A/mallard duck/Netherlands/08/2000 (H3N2) 95.1%
A/environment/West Java/KRW54/2012^a^	Karawang (West Java)	LPAI A (H3N8) Eurasian	A/duck/Siberia/100/2001 (H3N8) 89.9%	A/shoveler/Wisconsin/207/1978 (H6N8) 89.3%	A/duck/Mongolia/47/2001 (H7N1) 93.1%	A/goose/Eastern China/17/2010 (H6N6) 90.8%	A/environment/Korea/W266/2007 (H7N4) 96.9%	A/chicken/Nanchang/7-010/2000 (H3N6) 95.4%	A/swine/Kazakhstan/106/1985 (H3N6) 96.0%	A/mallard/Sweden/103/2005 (H2N9) 94.9%

^a^isolated during either human or suspect human case investigation

^b^isolated during live bird market surveillance

### Virulence and infectivity in mice

**Fig. 2:**
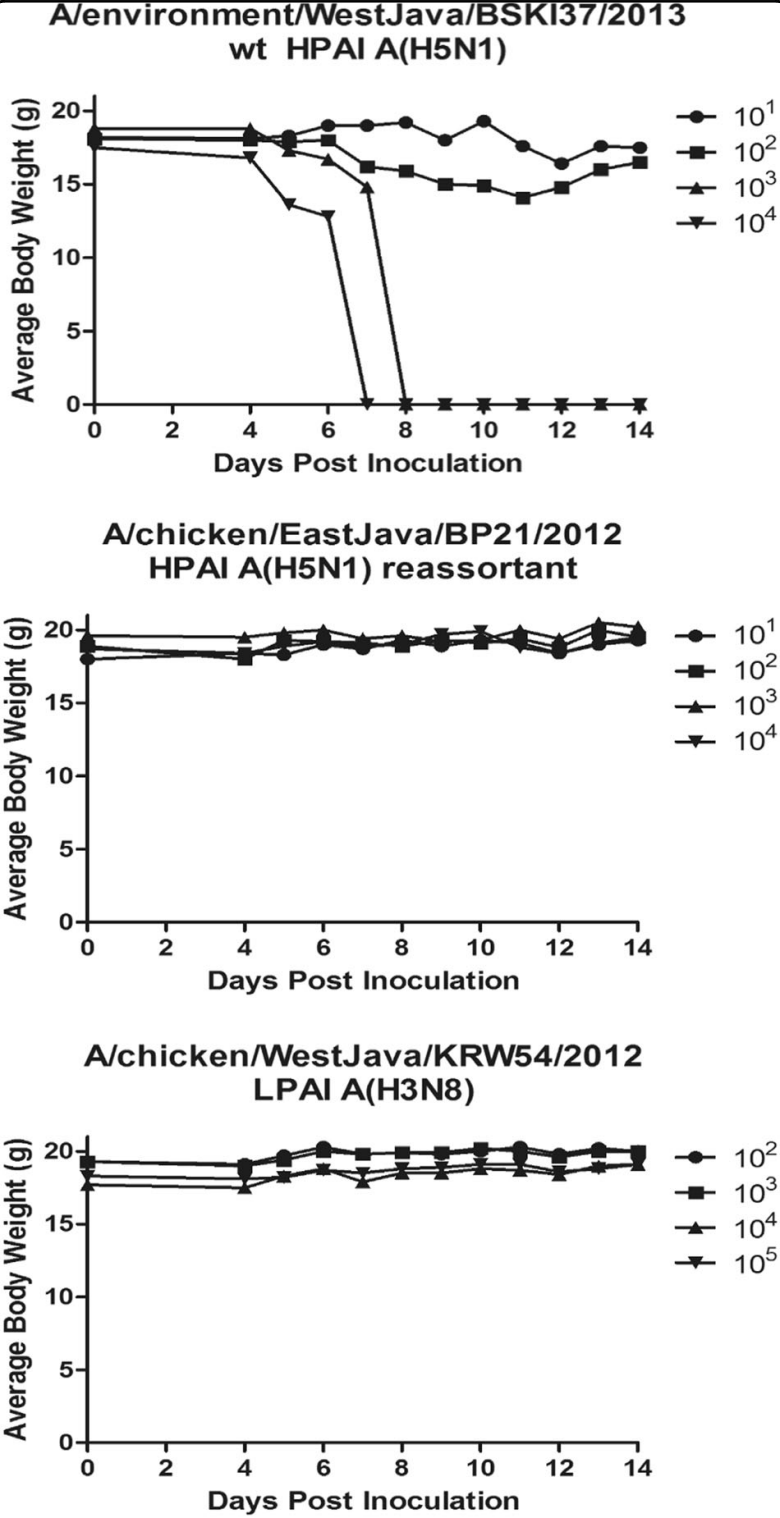
Weight change for mice inoculated intranasally with (**a**) A/environment/WestJava/BKSI37/2013 wt HPAI A(H5N1); **b** A/chicken/EastJava/BP21/2012 rt HPAI A(H5N1); or **c** A/chicken/WestJava/KRW54/2012 LPAI A(H3N8). The weight for five mice per dilution was determined until day 14. The mean for each time point is displayed. In Fig. 2a, dosage groups with mice that lost ≥ 25% of their pre-inoculation body weight were humanely euthanized; this is indicated with an average body weight of zero at the next time point after this experimental endpoint was reached. Dosage was measured in EID_50_/ml

**Fig. 3:**
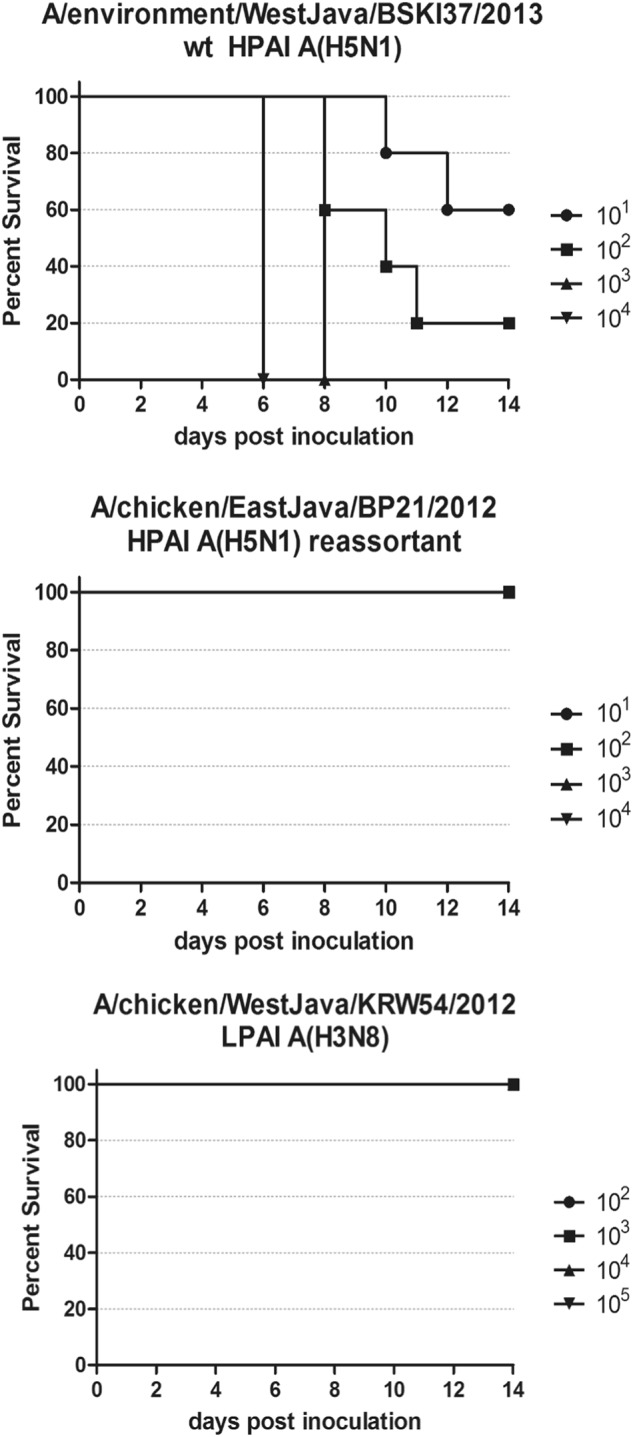
Lethality exhibited by mice infected with (**a**) wt HPAI A(H5N1), A/environment/West Java/BKSI37/2013. Infected mice were monitored for a 14 day experimental period for clinical signs of illness. Any mouse that exhibited a clinical sign of “4” or lost 25% or more of its body weight was humanely euthanized. Dosage was measure in EID_50_/ml. Groups of mice infected with either (**b**) rt HPAI A(H5N1), A/chicken/EastJava/BP21/2012 or (**c**) LPAI H3N8, A/chicken/WestJava/KRW54/2012 showed no mortality at any dose administered

We selected viruses for use in the mouse study that were representative of the wt HPAI A(H5N1) viruses (containing all H5N1 gene segments), the reassortant A(H5N1) virus and the LPAI A(H3N8) virus; these included A/environment/West Java/KRW54/2012 (LPAI A(H3N8), A/chicken/East Java/BP21/2012 (reassortant A(H5N1)) and A/environment/West Java/BKSI37/2013 (wt H5N1). The term “wild-type” indicates any virus studied that contains the typical constellation of H5N1 wild-type genes without reassortment. The groups of mice that were infected intranasally with 10^4^ and 10^3^ EID_50_ of the wt HPAI A(H5N1) virus, A/environment/West Java/BKSI37/2013, lost ≥ 25% of their pre-inoculation weight and/or showed clinical symptoms of infection within 6 and 8 days post-infection (dpi) and were humanely euthanized. The mice infected at lower doses (10^2^ and 10^1^ EID_50_) also experienced weight loss of ≥25% and symptoms of infection, resulting in variable survivability. Interestingly, those mice that survived infection at 10^2^ and 10^1^ EID_50_ also exhibited slight or no weight loss within 14 dpi and did not exhibit clinical symptoms, suggesting low levels of initial infection and/or viral replication (Fig. 2). Mice infected at every dosage level (10^1^–10^5^ EID_50_) with either the LPAI A(H3N8) virus (A/environment/West Java/KRW54/2012) or the reassortant HPAI A(H5N1) virus (A/chicken/East Java/BP21/2012) did not lose weight or exhibit clinical symptoms by 14 dpi. These mice, in fact, had a net weight gain of between 3 and 7%, irrespective of the inoculum dose (Fig. 2). A 100% survival rate, regardless of the dose, was observed for mice infected with either the LPAI A(H3N8) virus or the HPAI A(H5N1) reassortant virus. However, the group of mice infected with the wt HPAI A(H5N1) virus exhibited a dose-dependent survival curve, with 100% of the 10^4^ and 10^3^ EID_50_ infected mice either dying or meeting the experimental endpoint, while mice infected with 10^2^ and 10^1^ EID_50_ had 80% and 20% mortality, respectively (Fig. 3). The LD_50_ for the wt HPAI A(H5N1) virus was calculated to be 10^1.3^ EID_50_, while neither the reassortant nor the LPAI viruses reached an LD_50_ endpoint (i.e., LD_50_ was >10^4^ and >10^5^ EID_50_, respectively), Table 2.

**Table 2:** Mouse infectivity and virulence comparison

Virus	Subtype	MID_50_^a^	Viral titer (PFU) at 4 dpi at 10^3^ dose^b^				Mean days to death per dose^c^				LD_50_^d^
			Nose	Lung	Spleen	Brain	10^4^	10^3^	10^2^	10^1^	
A/environment/West Java/BKSI37/2013	wt HPAI A(H5N1)	10^2.2^	10^4.7^	10^6.5^	10^4.5^	10^3.5^	6	8	10.2	12.8	10^1.3^
A/chicken/East Java/BP21/2013	Reassortant HPAI A(H5N1)	>10^4.0^	<10^1^	10^3.0^	<10^1^	<10^1^	N/A	N/A	N/A	N/A	>10^4^
A/environment/West Java/KRW54/2012	LPAI A(H3N8)	10^4.8^	10^3.8^	<10^1^	<10^1^	<10^1^	N/A	N/A	N/A	N/A	>10^5^

^a^calculation based on the total number of infected tissues per virus per dosage level; reported as PFU per dose

^b^≤10^1^ pfu was the limit of detection of the assay

^c^N/A not applicable because no animals died during the experiment

^d^reported as PFU per dose

**Fig. 4:**
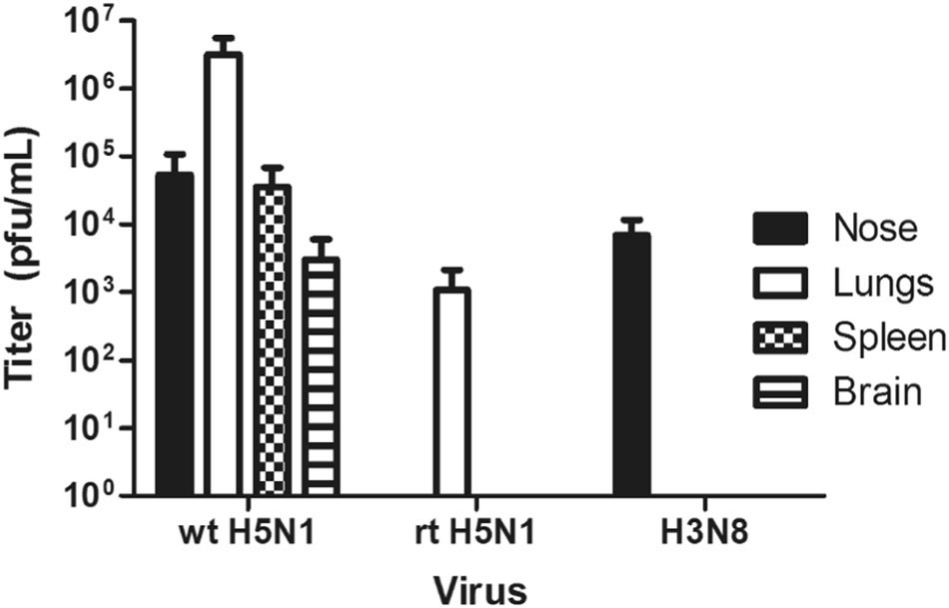
Average tissue viral titers from mice infected with 10^3^ EID_50_/ml. Tissue was collected from mice 4 days post infection, homogenized, and assessed for viral recovery by plaque assay using MDCK cells. wt HPAI H5N1: A/environment/West Java/BKSI37/2013, rt HPAI H5N1: A/chicken/East Java/BP21/2012, and H3N8: (LPAI) A/environment/West Java/KRW54/2012

Infectious virus was detected in all tissue samples from mice infected with the wt HPAI A(H5N1) virus, indicating a systemic infection. Infectious virus was detected in only the nose and lung tissue from mice infected with either the LPAI A(H3N8) or the HPAI A(H5N1) reassortant virus, A/chicken/East Java/BP21/2012. This suggests that these viruses were capable of infecting mice without further adaptation but incapable of causing a systemic infection (Fig. 4). The MID_50_ was calculated for each virus by determining the presence or absence of infectious virus in any one of four tissues that was collected at each dose at 4 dpi. The wt HPAI A(H5N1) virus MID_50_ was 10^2.2^ EID_50_, which was lower compared to that of either the reassortant A(H5N1) virus (MID_50_ > 10^4^ EID_50_) or the LPAI A(H3N8) virus (MID_50_ = 10^4.4^ EID_50_) (Table 2).

### Replication kinetics in embryonated chicken eggs and CEF cells

**Fig. 5:**
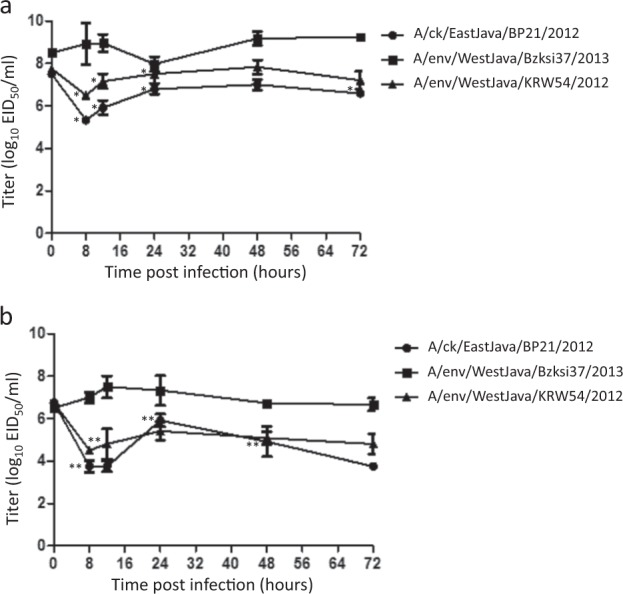
Replication kinetics of viruses in (**a**) embryonated chicken eggs (ECEs), and (**b**) chicken embryo fibroblast (CEF) cells. Log_10_ EID_50_/ml titers are shown as the mean (±SD) of independent experiments carried out in triplicate. Statistical variation between titers at each time point were calculated by student *t*-test and considered statistically significant at *p* = 0.01 (*) in ECEs and *p* = 0.25 (**) in CEFs. The wt HPAI A(H5N1) virus (A/environment/West Java/BKSI37/2013) is shown by circles, reassortant HPAI A(H5N1) virus (A/chicken/East Java/BP21/2012) by squares, and LPAI A(H3N8) virus (A/chicken/West Java/KRW54/2012) by diamonds

The replication kinetics of each virus was evaluated in both CEF cells and ECEs via the comparison of the mean viral titers at specific timepoints post-inoculation (Fig. 5). In both substrates, the non-reassortant HPAI A(H5N1) virus (A/environment/WestJava/BKSI37/2013) reached higher titers at earlier time points, with an average peak titer of 10^7.5^ EID_50_/ml in CEF cells and 10^9.25^ EID_50_/ml in ECEs (Fig. 5). Both the reassortant HPAI A(H5N1) (A/chicken/EastJava/BP21/2012) and the LPAI A (H3N8) (A/environment/WestJava/BKSI37/2013) virus had an initial decrease in their titer in both substrates, with the peak titers at each time point that was measured being lower than those of the non-reassortant HPAI virus. While the post-inoculation titers of the reassortant HPAI virus were not significantly different compared to those of the LPAI virus, they were generally reduced by comparison at the majority of the time points that were tested in both substrates (Fig. 5).

### Amino acid sequence comparison

Analysis of the amino acid substitutions present in each viral protein was undertaken to predict the critical phenotypic properties of each Indonesian virus included in this study (Table 3). The HA amino acid sequence in all of the five HPAI viruses possessed a highly pathogenic avian influenza virus multi-basic cleavage site motif, PQRESRRKKR↓G. Analysis of the H3 HA gene of A/environment/West Java/KRW54/2012 revealed a cleavage site, PEKQTR↓G, that is typical of LPAI viruses (Table 3). All of the five HPAI viruses had four putative glycosylation sites at positions 10, 11, 24, and 165 in the HA1 protein, and A/chicken Bengkulu/RJL24/2013 and A/environment/West Java/BKSI37/2013 had an additional glycosylation site in HA1 at position 154. No substitutions were identified in the HA of the LPAI virus that were predicted to alter the viral phenotype. The five HPAI A(H5N1) viruses all had an NA protein that contained a deletion of 20 amino acids in the stalk region (residues 49–68). This NA feature is conserved among HPAI A(H5N1) viruses and has been shown to enhance virulence in mice^15,16^.

**Table 3:** Molecular comparison of 2012–2013 viruses from Indonesian live bird markets

Strain	Type	HA		NA	PB2			PB1-F2	PA	M1		M2	NS1			
		CS^a^	156^b^	Del 49-68^c^	627^d^	701^d^	526^d^	87–90^d^	149^d^	30^d^	215^d^	27^d^	42^d^	Del 80-84^c^	92^d^	PDZ ligand domain
A/chicken/Bengkulu/RJL24/2013	HPAI A(H5N1) wt	PQRES**RRKKR**	T	YES	E	D	R	YES	S	D	A	A	S	YES	E	ESEV
A/environment/West Java/BKSI37/2013	HPAI A(H5N1) wt	PQRES**RRKKR**	T	YES	E	D	R	YES	S	D	A	A	S	YES	E	ESEV
A/muscovy duck/East Java/SB29/2012	HPAI A(H5N1) Reassortant	PQRES**RRKKR**	A	YES	E	D	K	YES	S	D	A	A	S	NO	D	ESEV
A/muscovy duck/East Java/LM47/2012	HPAI A(H5N1) Reassortant	PQRES**RRKKR**	A	YES	E	D	K	YES	S	D	A	A	S	NO	D	ESEV
A/chicken/East Java/BP21/2012	HPAI A(H5N1) Reassortant	PQRES**RRKKR**	A	YES	E	D	K	YES	S	D	A	A	S	NO	D	ESEV
A/environment/West Java/KRW54/2012	LPAI A(H3N8)	PEKQT**R**GLF	K	NO	E	D	K	YES	S	D	V	I	S	NO	D	ESEV

^a^*CS* cleavage site

^b^Mature H5 HA numbering

^c^NA deletion and NS1 deletion numbering is relative to A/goose/Guangdong/1/1996

^d^Internal protein numbering is relative to A/Vietnam/1203/2004

The amino acid sequences of the six internal genes of each virus were also analyzed and compared for the presence of molecular features of interest (Table 3). The PB2 in all of the analyzed viruses contained the avian-specific host markers 627E and 701D. Other substitutions that were found only in the viruses from West Java (all wild type HPAI viruses) included 89 V, 391E, 477 G, and 526 R. The substitution 526 R has been shown to dramatically affect the viral phenotype by increasing the polymerase activity^17^. The PB2 in the viruses from East Java and also in the LPAI A(H3N8) virus possessed the following substitutions: 89 V, 309D, 339 K, 477 G, 495 V, 627 E, and 676 T. These substitutions comprise a constellation of changes that, even in the absence of the 627 K substitution, have been shown to enhance the polymerase activity and increase virulence in mice^18^. Most notably, neither the LPAI A(H3N8) nor the East Java viruses had the K526R substitution. The PB1 protein in all of the analyzed viruses was the full-length PB1-F2 version, which has been demonstrated to affect virulence in mice^19^. The PA protein in all of the viruses had a serine at position 149 (149 S). The MP in all of the analyzed HPAI A(H5N1) viruses had the same M1 and M2 amino acid substitutions, i.e., either 30D or 215 and 27 A, respectively. The LPAI A(H3N8) virus had contained only the M1 30D amino acid substitution The NS1 protein in all of the analyzed viruses contained the 42 S substitution, while only the West Java viruses contained the deletion of residues 81–86. All of the analyzed viruses had a PDZ ligand motif in ESEV, which is not only conserved in contemporary H5N1 viruses but also associated with increased virulence in mice^20^. The NP protein in all of the viruses lacked any amino acid substitutions that have been experimentally identified as affecting viral phenotype (Table 3).

## Discussion

Six viruses collected from LBMs from both the islands of Java and Sumatra, Indonesia, were obtained either as part of an investigation of human case or through active surveillance. The HA and NA genes contained in five of the six isolated viruses were found to be phylogenetically grouped with other clade 2.1.3.2a A(H5N1) viruses from Indonesia. These viruses were isolated from LBMs that were representative of others within the entire island of Java (provinces of Bekasi, Gresik, Lamongan, and Surabaya), with one isolate obtained from Bengkulu in the eastern province of Sumatra. The sixth virus was collected from the Karawang District on the island of Java and was revealed to be of the LPAI A(H3N8) subtype based on the BLAST and phylogenetic analysis. The PB2, PB1, and NS genes from three A(H5N1) viruses from East Java were found to be grouped with those from the West Java LPAI A(H3N8) virus, while the other internal genes suggested a close evolutionary relationship with other HPAI A(H5N1) viruses from Indonesia. However, the HPAI A(H5N1) viruses collected in West Java did not contain a similar constellation of genomic reassortments. To assess the pandemic potential of newly emerging viruses, it is imperative that we can determine the degree of infectivity of avian influenza A(H5N1) viruses in various mammalian species. Although the ferret model of infection is also a suitable model to assess the pathogenicity of influenza viruses, the mouse model has been used for many years due to the susceptibility of mice to HPAI H5N1 viruses without prior virus adaptation as well as a significantly decreased financial and laboratory burden, allowing for the use of large numbers of animals to ensure reproducibility. In the mouse model of infection, the non-reassortant HPAI clade 2.1.3.2a virus induced the typical signs of infection that result from an HPAI A(H5N1) virus (e.g., hunched posture, ruffled fur, lethargy, and anorexia leading to extreme weight loss)^21^. The LD_50_ for this virus was 10^1.3^ EID_50_, which is similar to that of many other A/goose/Guangdong/1/1996-lineage A(H5N1) viruses^22–24^. This virus has proved to have greater lethality when compared to that of viruses isolated from animals in Indonesia^25^. Mice infected with either the LPAI A(H3N8) or the reassortant clade 2.1.3.2a viruses, however, did not show clinical symptoms of infection, and neither group experienced significant mortality. Only the non-reassortant HPAI A(H5N1) virus reached a high titer in the respiratory tract and was recovered from multiple organs, including the brain and spleen, indicating a systemic viral infection. Although we were able to isolate the virus from both the nose and the lung tissues of mice infected with the reassortant and LPAI viruses at the highest doses tested, the MLD_50_ values for both were greater than 10^4^ EID_50_, indicating a significantly attenuated infectivity phenotype compared to the non-reassortant virus. Thus, the acquisition of the PB2, PB1, and NS genes from the LPAI A virus by an HPAI A(H5N1) virus appeared to be sufficient to attenuate the infectivity of A/chicken/East Java/BP21/2012 in the mouse model.

While the assessment of viral fitness in a mouse model of infection is important for understanding how these viruses replicate in a mammalian host, we also characterized the replication capacity of these viruses in two in vitro avian host systems. All of the viruses were able to grow in both CEF cells and ECEs, but only the non-reassortant HPAI A(H5N1) strain (A/environment/WestJava/BKSI37/2013) exhibited replication kinetics typical of HPAI A(H5N1) viruses, with post-infection titers at most time points that were 1-2 logs above that of the starting inoculum. Both the reassortant HPAI A(H5N1) and the LPAI A(H3N8) strains (A/chicken/EastJava/BP21/2012 and A/environment/WestJava/KRW54/2012, respectively) exhibited an attenuated ability to replicate in both avian systems in comparison to the non-reassortant virus, as evidenced by decreased titers at the early time points and peak titers that were lower than those of the initial inoculum. Interestingly, the reassortant virus also reached lower titers at a majority of the time points tested when compared to the LPAI virus, indicating a slightly lower replication capacity due to the reassortment. The attenuation of the infectivity of the reassortant A(H5N1) virus in both mammalian and avian systems suggests that the acquisition of the internal genes from the LPAI A virus was sufficient to attenuate replication despite the continued presence of the multi-basic cleavage site in the HA protein.

Each of the HPAI A(H5N1) viruses from Indonesia described in this study contained an HPAI multi-basic cleavage site motif. This motif has been demonstrated to facilitate the dissemination of the virus to the extra-pulmonary organs in ferrets and to act as a host-specific virulence factor^26,27^. The HA glycoprotein also determines the viral host range by mediating binding to host cell sialic acid receptors^28,29^. The results of our analysis showed that there were no changes to the receptor binding sites of the HA gene in the HPAI A(H5N1) viruses in this study, suggesting that these viruses preferentially bind avian α-2,3 SA. The location and number of glycosylation sites in the H5 HA glycoprotein have also been demonstrated to not only affect receptor binding specificity but also mammalian virulence. Within the HPAI viruses described in this study, the HA mutation T156A was identified in all three of the reassortant viruses. The T156A substitution, which results in the loss of an N-linked glycosylation site, has been linked to an increase in binding to α2,6 sialoglycan cellular receptors and is associated with increased transmission in a guinea pig model of influenza infection^30,31^. Combined with other HA mutations, loss of glycosylation at this position has been shown to increase viral titers obtained from ferret nasal turbinates^32^ and to increase pathogenicity, systemic spread, and pulmonary inflammation in mice^33^. By reviewing surveillance data, Russell et al. were able to surmise that the T156A substitution at the HA glycosylation site may not be stable in viruses isolated from an avian host, suggesting that the mutation is under little selective pressure in hosts^34^. Although a reassortant virus containing this substitution was not found to be lethal in mice, it is possible that T156A slightly enhanced its lung infectivity, even though the virus did not have any other molecular features contributing to a virulent phenotype.

Each of the HPAI A(H5N1) viruses described in this study contained a deletion of 20 amino acids in the stalk region of the NA protein (residues 49-68). This mutation is conserved in all HPAI A(H5N1) viruses^15,16^ and, as nearly all of the human H5N1 infections that have been documented so far have been acquired from close interactions with infected poultry, all of the human isolates have this deletion as well. This deletion has been reported to increase virulence in mice, and in vitro studies demonstrated that it reduced the ability of the virus to both elute from agglutinated erythrocytes and penetrate mucus from cells of mammalian origin^15,35^. The stalk deletion mutation was also shown to change the tissue tropism from the intestine to the respiratory tract in chickens, as well as inhibit viral replication in ducks^36,37^. These observations support the idea that, in addition to enhancing the virulence of HPAI A(H5N1) viruses in the mouse model, this stalk deletion is a viral adaptation that originates from low-pathogenic strains typically found in waterfowl and land-based birds^16,36^.

The polymerase complex, which is made up of the PB2, PB1, and PA subunits, is utilized by the influenza virus to replicate and transcribe the viral genome in the cell nucleus. Mutations in the polymerase subunits that increase the transcriptional activity are essential for avian influenza viruses to adapt to a mammalian host. Although several mutations have been identified as markers of adaptation to mammals, the best characterized of these mutations is PB2-E627K^23,38–41^, which has been shown to increase viral replication, polymerase activity, and virulence in mammalian cells^42^. Another commonly described marker of mammalian adaptation is PB2-D701N^18,43^, which is a substitution that has been shown to enhance the viral replication efficiency and transmission in several mammalian models of influenza infection^18,31,39^. Neither the HPAI viruses or the LPAI A(H3N8) virus described in this study had the PB2-627K or 701N markers. Instead, all had PB2-627E and 701D, which are typical of avian-adapted viruses. Interestingly, the wt HPAI A(H5N1) viruses described in this study contained PB2-526R, while the reassortant and LPAI viruses did not. Although the PB2-K526R mutation is infrequently identified in viruses originating from poultry, it appears in the majority of viruses that have been isolated from human H5N1 cases in Indonesia^17^. This suggests that, although none of the viruses in this study contained either 627 K or 701 N, viruses with PB2-526R may be more adapted to mammalian replication than viruses without this change. Although the LPAI A(H3N8) virus failed to cause disease in mice, its PB2 gene contains a constellation of amino acid substitutions that, when present in combination with 627E, may enhance its viral polymerase activity and virulence in mice^18^.

Each of the HPAI A(H5N1) viruses isolated from Indonesia had an intact PB1-F2 protein (87–90 amino acids), regardless of their reassortment status. PB1-F2 is a pro-apoptotic mitochondrial protein that induces host immune cell death and has an immunomodulatory role that mediates virulence in the mouse model^19^. A functional PB1-F2 protein is found in more than 80% of all influenza viruses, regardless of their origin or subtype^44^. The implications of this finding remain unknown. The only mutation in PA that was found in all of the analyzed HPAI A(H5N1) viruses that affected the phenotype of the virus was 149 S, which is one of a combination of mutations (P149S, R266H, K257I, T515S) that together were found to decrease the activity of the polymerase complex in mammalian cells^45^. This finding suggests that all of the HPAI A(H5N1) viruses included in this study retained normal polymerase functionality. The M1 protein from the HPAI A(H5N1) Indonesian viruses was found to be related to that from other H5N1 viruses and also to have the amino acid substitutions M30D and T215A. These two substitutions have been shown to have a combinatory effect on the degree of virulence in mice^46^, although the underlying mechanism remains unknown. Additionally, all of the HPAI viruses from Indonesia that were included in this study contained an amino acid substitution in the M2 transmembrane domain of V27A that has been reported to reduce the susceptibility of several viral subtypes to amantadine^47–49^, suggesting that these viruses may also exhibit some degree of resistance. The NS1 protein from all of the analyzed HPAI A(H5N1) viruses from Indonesia contained multiple mutations and/or sequence motifs that have been experimentally shown to enhance virulence in mice. The viruses isolated from the specimens from Bengkulu and West Java possessed an NS gene derived from other HPAI A(H5N1) viruses, while the reassortants from East Java and the LPAI A(H3N8) virus possessed an NS gene derived from an LPAI progenitor virus. All of the viruses, regardless of their reassortment status, had a serine at position 42 (NS1-42S) as well as four C-terminal residues that form a PDZ domain ligand of the X-S/T-X-V type in ESEV. The presence of both the NS1-42S substitution and the PDZ ligand motif were reported to increase virulence in a mouse model of influenza infection^20,50^. Other NS1 markers of enhanced virulence were only noted in the non-reassortant, wt viruses isolated from Bengkulu and West Java. Each of the wt viruses also contained a deletion of residues 80–84 and a glutamic acid at position 92 (NS1-92E); the presence of both of these alterations are associated with increased virulence in a mouse model and may play a role in the differential virulence of the wt versus that of the reassortant viruses^51,52^.

In Indonesia, surveillance for human infections and outbreaks in birds continues to provide epidemiological and virological information about viral evolution and the circulation of clades and mutations that may be predictive of the loss or gain of mammalian adaptations^1,53^. Although recent Indonesian HPAI A(H5N1) sequence data point to the predominance of the HPAI A(H5N1) clade 2.3.2.1c (Fig. 1a), it remains plausible that molecular changes, such as those described herein, have led to a decrease in the number of human infections detected in Indonesia. Surveillance in birds is conducted in live bird markets, poultry collection yards, and farms^5^, while virological surveillance of human infections is conducted at 26 sentinel healthcare centers and 6 hospitals throughout Indonesia^54,55^. In this study, we reported on reassortant avian influenza viruses that were isolated during investigations of outbreaks associated with human A(H5N1) virus infection reports, as well as during routine influenza virus surveillance in birds in Indonesia. In other countries, virological surveillance has provided an understanding of the spatiotemporal evolution of avian influenza viruses^56^. In Nigeria, within one year of the first detection of the circulation of the H5N1 virus, reassortant strains were isolated and rapidly became dominant in poultry populations^57^. In Bangladesh, reassortant H5N1 viruses containing an H9N2-PB1 gene were detected in poultry, which likely originated from the co-circulation of the H5N1 and H9N2 viruses^58^. The results described herein highlight the fact that the co-circulation of different viruses in the same susceptible population can give rise to novel reassortants. Furthermore, there is evidence that reassorted viruses may display vastly different phenotypes than either parental strain and may cause human infection. A study in China found that in a farm with co-circulating H7N9 and H9N2 viruses, human infection was able to occur due to a genotype resulting from natural reassortment^59^. In addition to naturally occurring reassortant influenza viruses, studies involving reverse genetics have demonstrated that avian influenza viruses can acquire human genes. In a study that assessed reassortment between avian H5N1 and human H3N2 viruses, 22 out of the 75 reassortant H5 viruses were more pathogenic than their parental counterparts, while others were attenuated ^60^. These results, as well as those of the current study, highlight the need for ongoing surveillance in both humans and birds to assess viral evolution and detect the generation of reassortant viruses with epizootic and zoonotic potential.

## Electronic supplementary material

Figure S1 (a) and (b): Phylogenetic tree of (a) HPAI A(H5N1) and (b) LPAI A virus NA genes. Figure S2 (a-f): Phylogenetic tree of HPAI A(H5N1) and LPAI A virus internal genes; PB2(a), PB1(b), PA(c),
