# Supplementary material for: Attenuation of highly pathogenic avian influenza A(H5N1) viruses in Indonesia following the reassortment and acquisition of genes from low pathogenicity avian influenza A virus progenitors
Source: Emerg Microbes Infect. 2018 Aug 22;7:147. doi: 10.1038/s41426-018-0147-5 (PMC6104089; doi:10.1038/s41426-018-0147-5)

Table S1. Specimens collected and viruses positive from the four provinces, Indonesia 2012-2013

Province/ District	Investigation type	Date of collection	Cloacal specimens	Fecal specimens	Environmental surface specimens	A/H5N1 virus positive pools	Other influenza viruses	Viruses characterized
West Java, Karawang	Human case	3-5 July, 2012	117	36	24	5	H3N8 (n=1)	A/environment/West Java/KRW54/2012
Bengkulu, Rejang Lebung	Human case	6-8 March, 2013	72	0	31	1	0	A/chicken/Bengkulu/RJL24/2013
West Java, Bekasi	Human case	25 June, 2013	11	0	68	5	0	A/Environment/West Java/Bksi37/2013
East Java, Gresik	Market surveillance	5 November, 2012	23	0	30	3	0	A/Chicken/East Java/BP21/2012
East Java, Surabaya	Market surveillance	8-9 November, 2012	24	0	29	1	0	A/Muscovy duck/East Java/SB29/2012
East Java, Lamongan	Market surveillance	7 November, 2012	57	0	30	4	0	A/Muscovy Duck/East Java/LM47/2012

Supplemental Figure 1 (a) and (b): Phylogenetic tree of (a) HPAI A(H5N1) and (b) LPAI A virus NA genes. Viruses characterized herein are identified as follows: Indonesia H5N1 are colored red, Indonesia reassortant H5N1 are colored blue, Indonesia LPAI are colored green, and all virus further utilized for *in vivo* studies are underlined. Bootstrap values calculated following 1000 replicates are shown above each branch.

Figure S1(a)
N1 gene

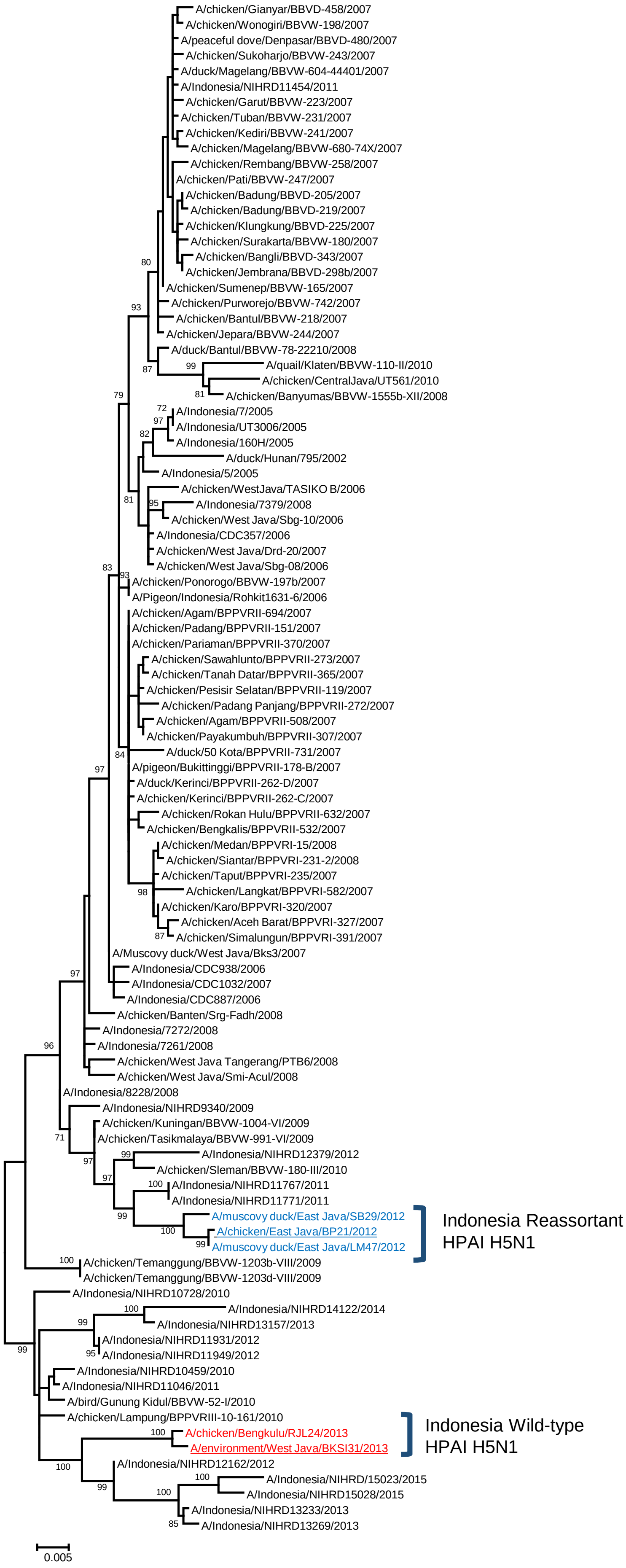

Figure s1(b)
N8 gene

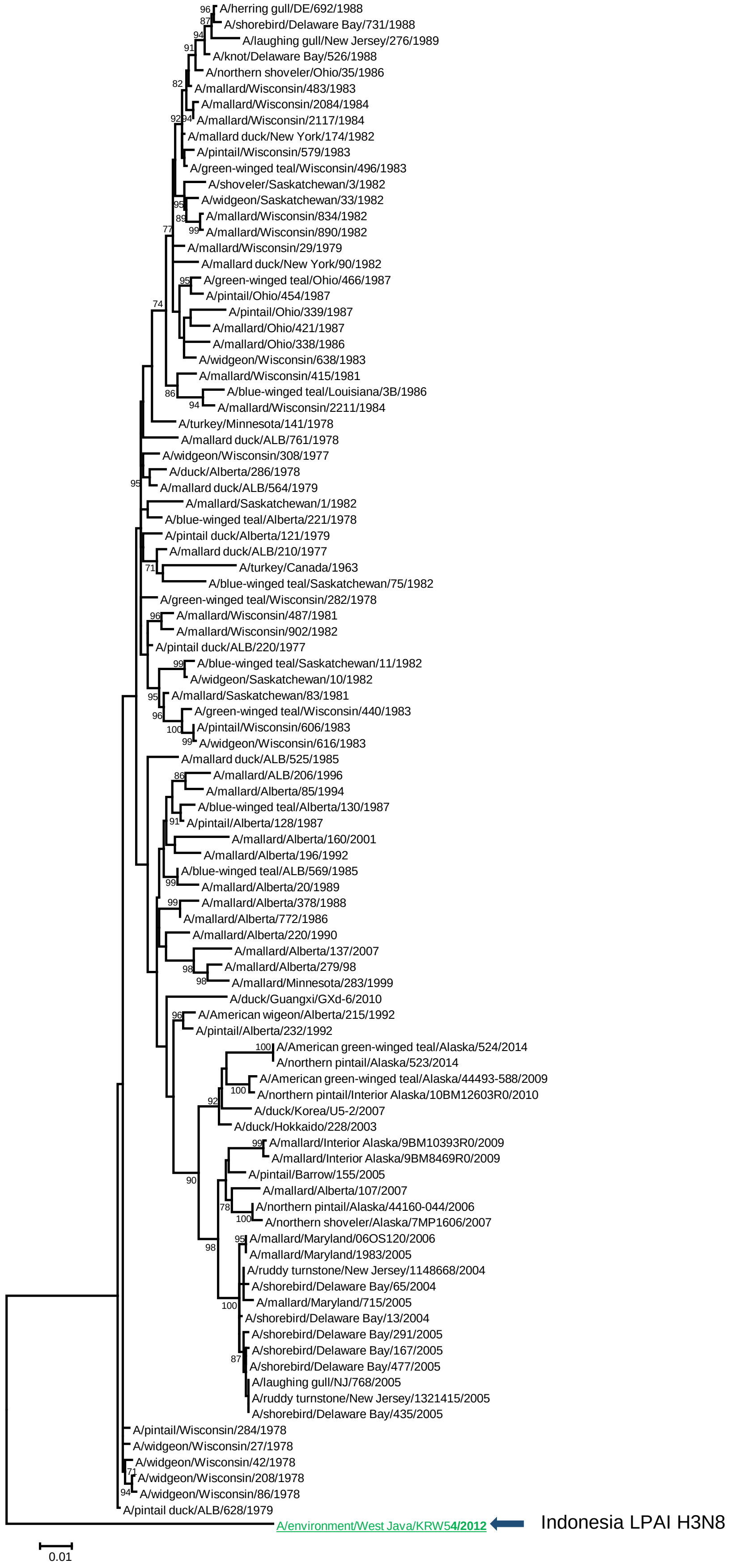

Supplemental Figure 2 (a-f): Phylogenetic tree of HPAI A(H5N1) and LPAI A virus internal genes; PB2(a), PB1(b), PA(c), NP(d), M(e), NS(f). Viruses characterized herein are identified as follows: Indonesia H5N1 are colored red, Indonesia reassortant H5N1 are colored blue, Indonesia LPAI are colored green, and all virus further utilized for *in vivo* studies are underlined. Bootstrap values calculated following 1000 replicates are shown above each branch.

Figure S2(a)
PB2 gene

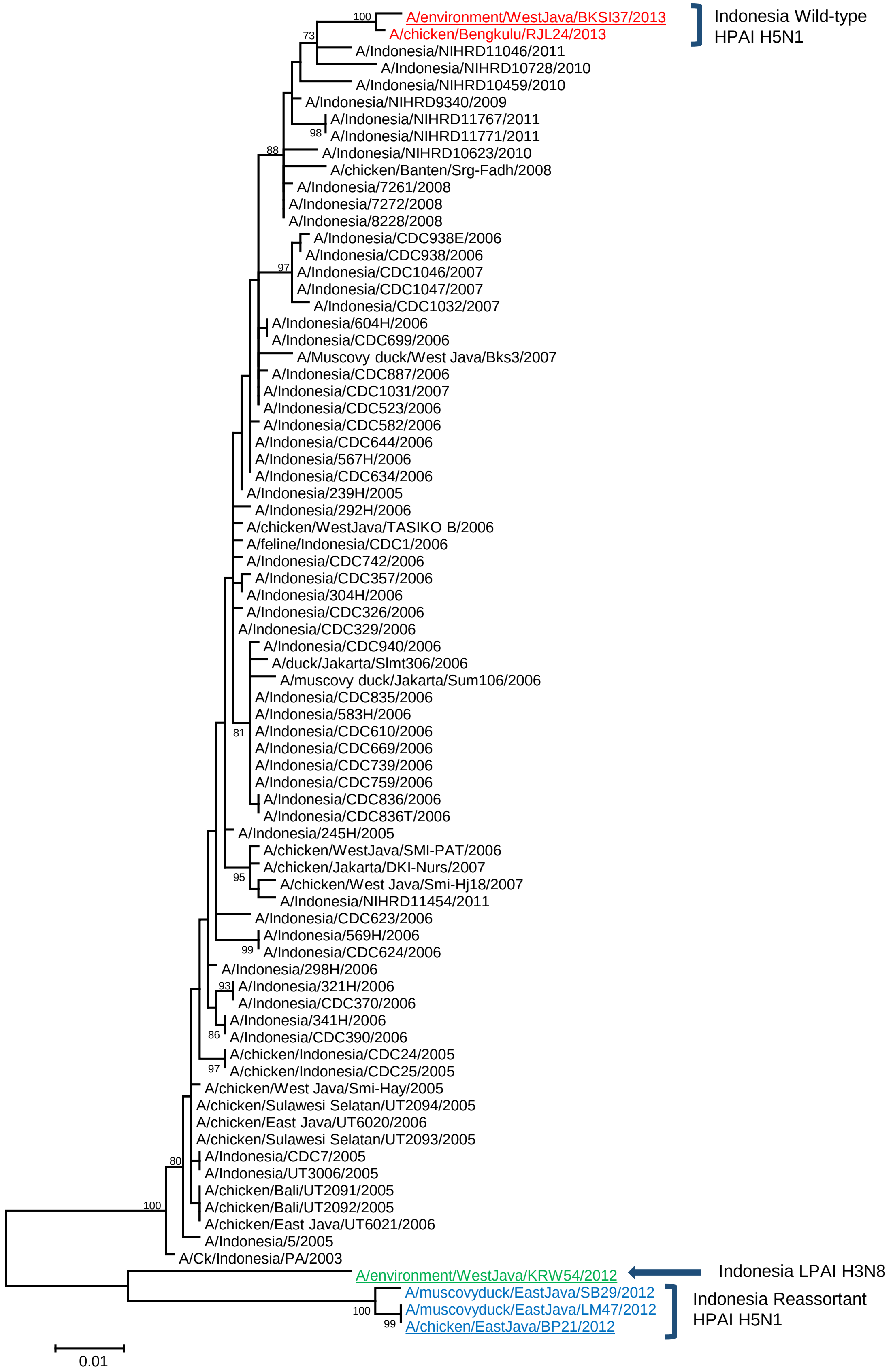

Figure S2(b)
PB1 gene

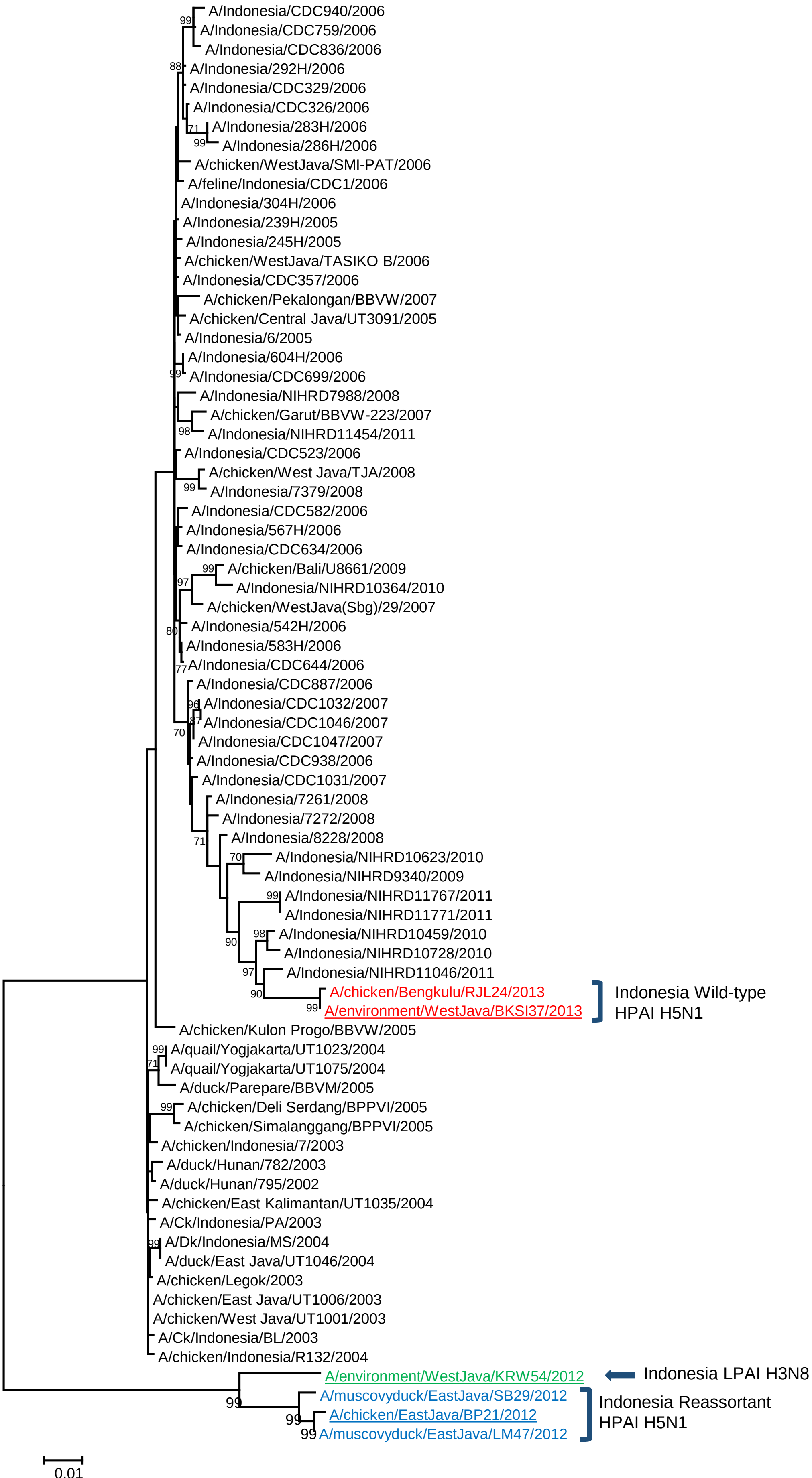

Figure S2(c)
PA gene

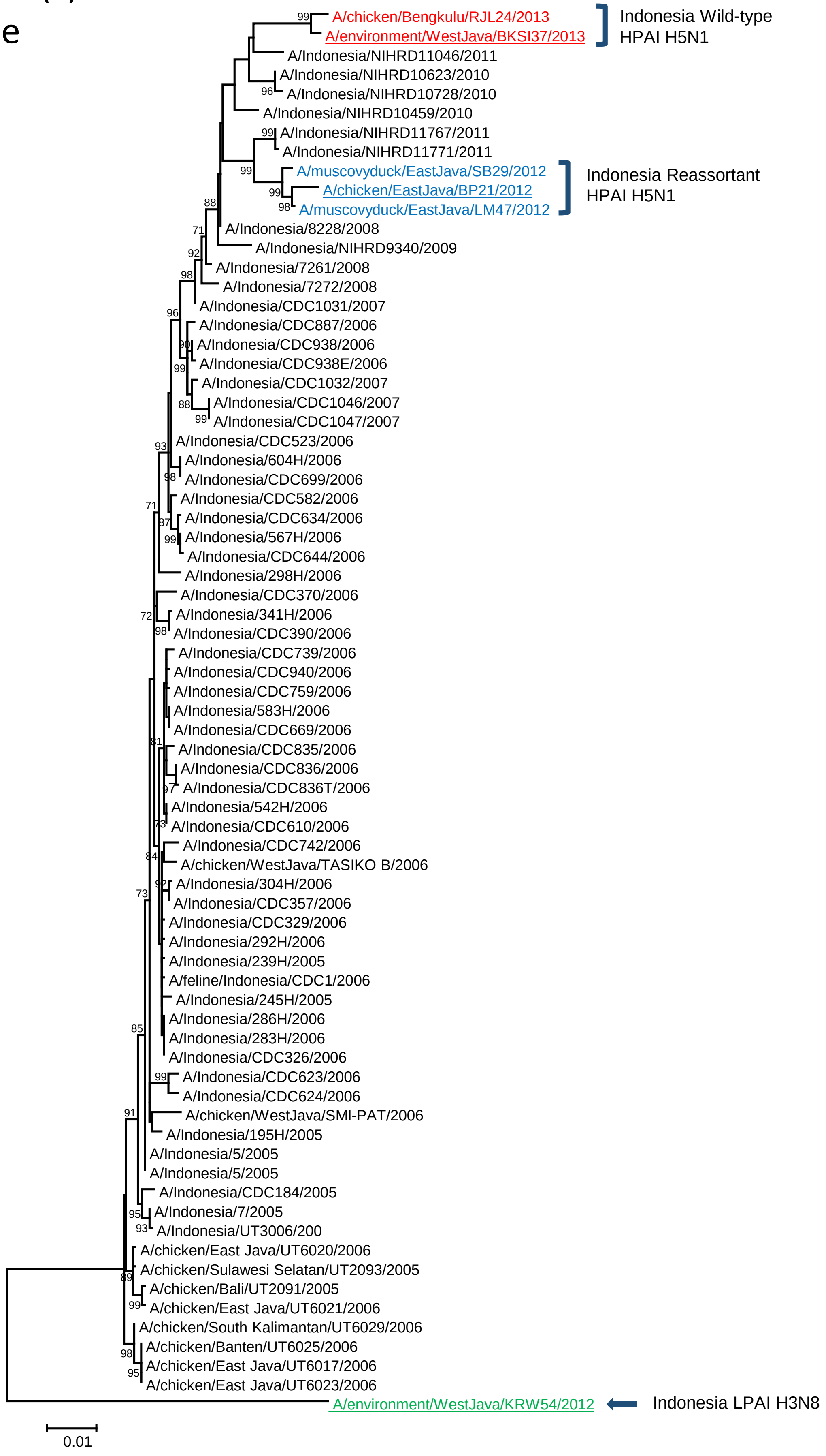

Figure S2(d)
NP gene

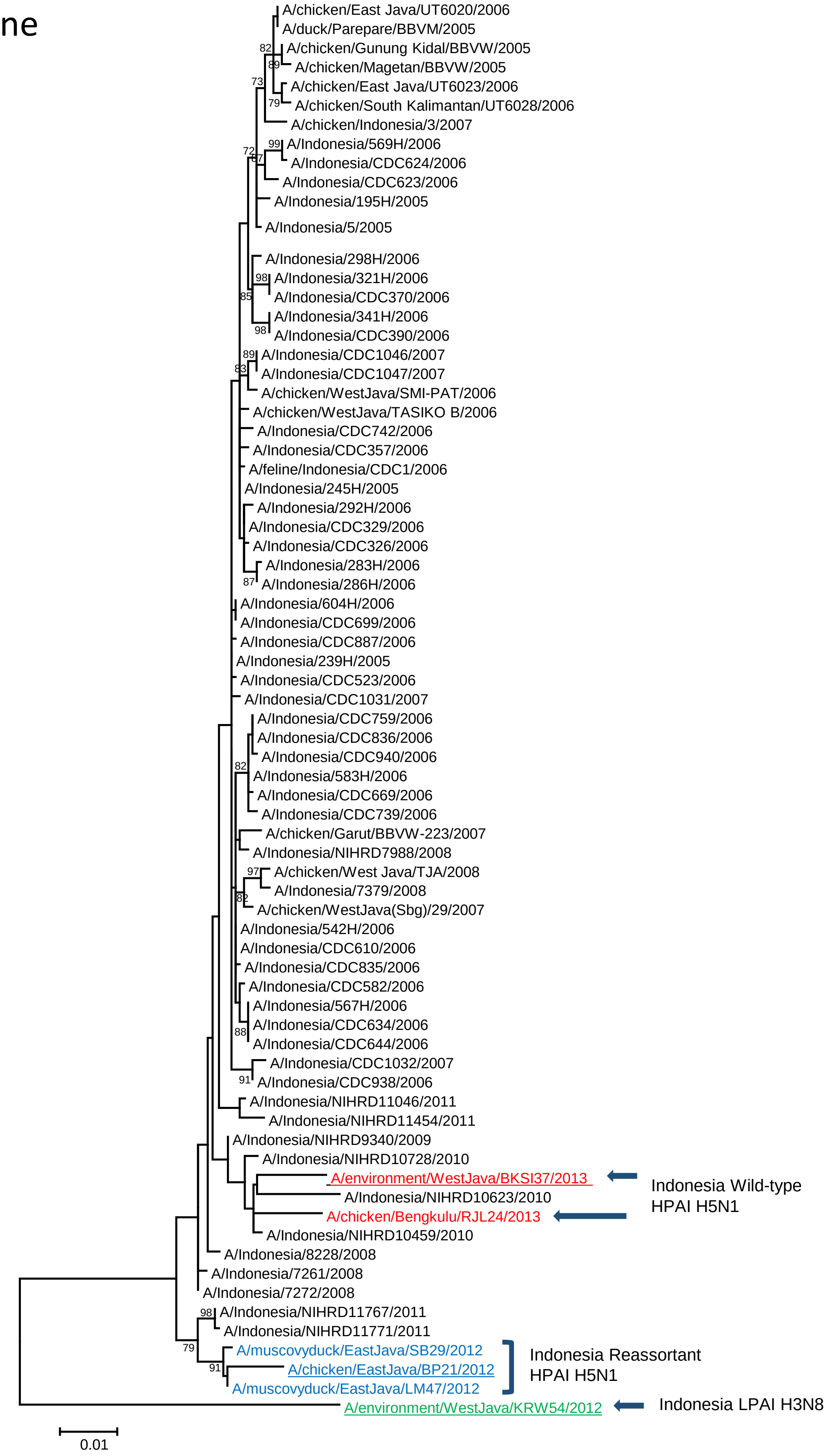

Figure S2(e)
M gene

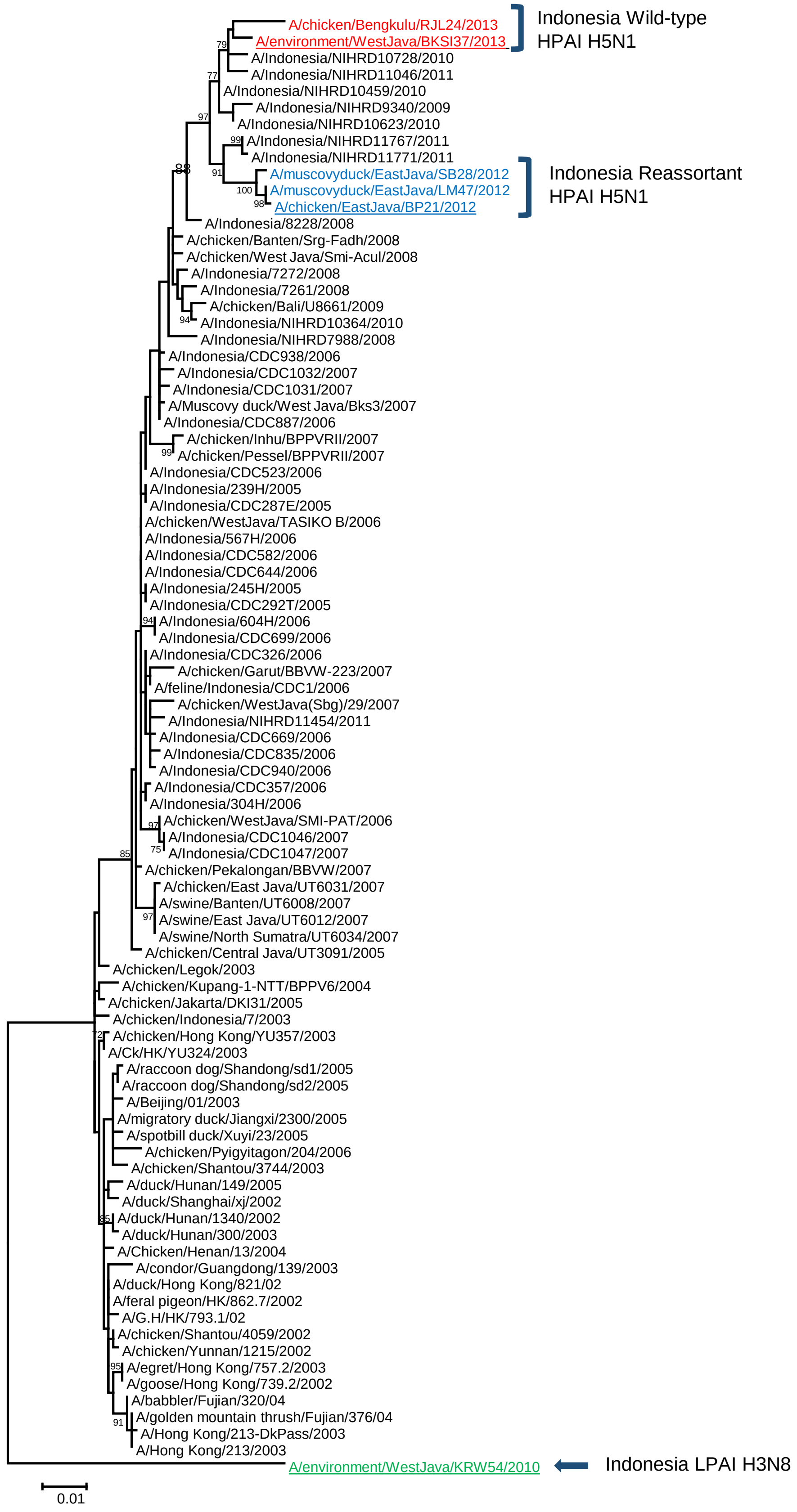

Figure S2(f)
NS gene

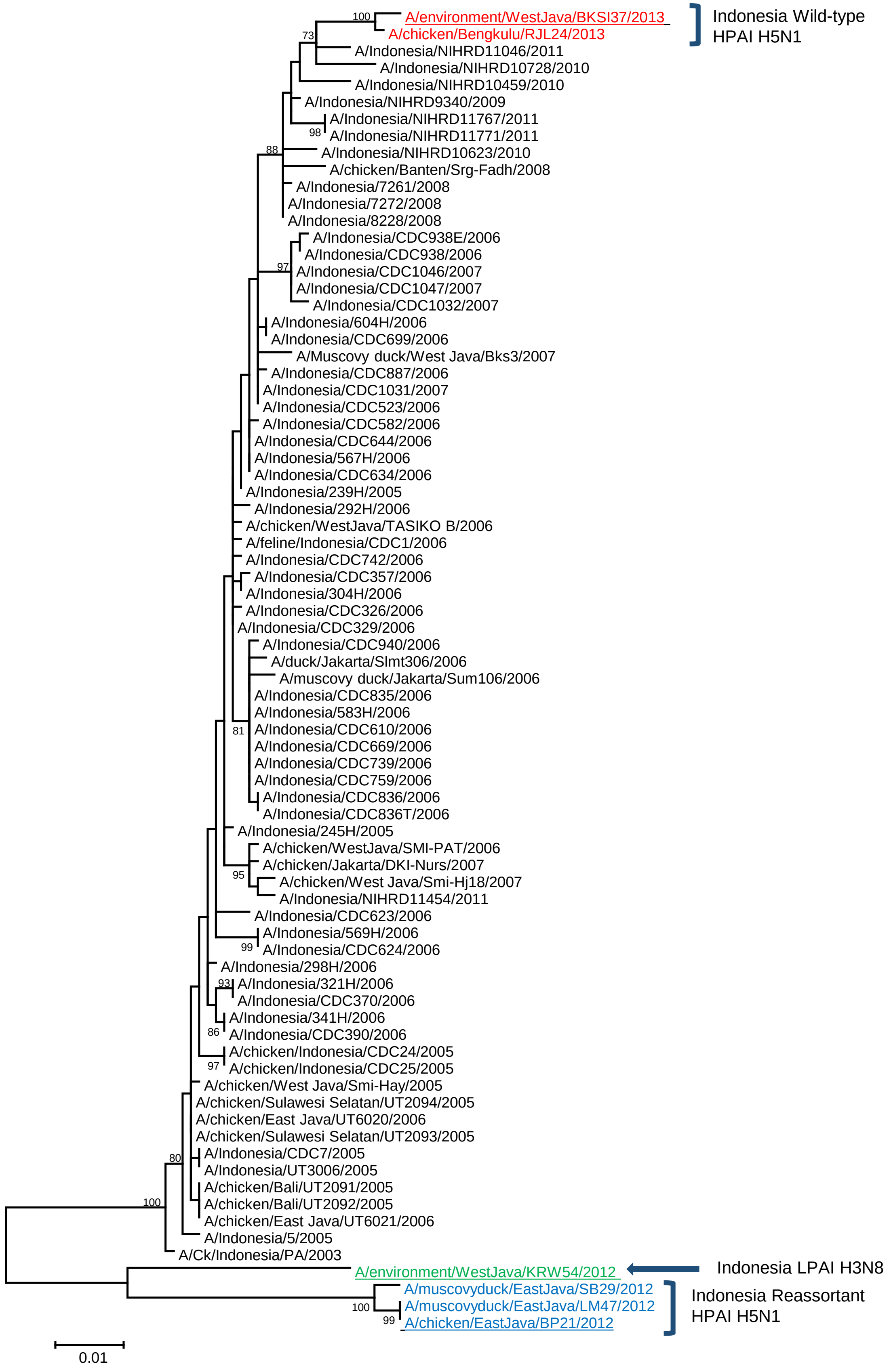

Supplement: Supplementary file 1 — Figure S1 (a) and (b): Phylogenetic tree of (a) HPAI A(H5N1) and (b) LPAI A virus NA genes. Figure S2 (a-f): Phylogenetic tree of HPAI A(H5N1) and LPAI A virus internal genes; PB2(a), PB1(b), PA(c), [file 41426_2018_147_MOESM1_ESM.pdf]
